# IL-27-Induced Gene Expression Is Downregulated in HIV-Infected Subjects

**DOI:** 10.1371/journal.pone.0045706

**Published:** 2012-09-25

**Authors:** Christina Guzzo, Wilma M. Hopman, Nor Fazila Che Mat, Wendy Wobeser, Katrina Gee

**Affiliations:** 1 Department of Biomedical and Molecular Sciences, Queen’s University, Kingston, Ontario, Canada; 2 Department of Community Health and Epidemiology, Queen’s University, Kingston, Ontario, Canada; 3 Division of Infectious Diseases, Department of Medicine, Queen’s University, Kingston, Ontario, Canada; 4 School of Health Sciences, Universiti Sains Malaysia, Kelantan, Malaysia; University of London, St George’s, United Kingdom

## Abstract

**Objective:**

To characterize the effect of HIV infection on IL-27-induced gene expression.

**Design:**

During HIV infection, cytokine expression and function become deregulated. IL-27 is an important modulator of inflammatory responses. Interestingly, IL-27 can inhibit HIV replication in T cells and monocytes, implicating IL-27 as a potential adjunct to anti-viral treatment. Our previous work demonstrated that circulating HIV may suppress IL-27 expression, therefore, this study, in continuation of our previous work, aimed to understand how HIV affects expression levels of the IL-27 receptor and downstream functions of IL-27.

**Methods:**

Peripheral blood mononuclear cells (PBMC) were isolated from whole blood of HIV negative and HIV positive (viremic) individuals to assess IL-27-induced gene expression by flow cytometry and ELISA. PBMC were also processed for monocyte enrichment to assess IL-27 receptor expression by flow cytometry and real-time PCR.

**Results:**

Expression of the IL-27 receptor subunit, gp130, was upregulated in response to IL-27 in HIV negative individuals, however, in HIV positive individuals, this IL-27 response was diminished. Furthermore, we observed downregulation of IL-27-induced IL-6, TNF-α, and IL-10 expression in HIV positive subjects.

**Conclusion:**

In HIV infection, IL-27-induced gene expression was impaired, indicating HIV-mediated dysregulation of IL-27 functions occurs during HIV infection. This study provides evidence for new viral pathogenic mechanisms contributing to the widespread impairment of immune responses observed in HIV pathogenesis.

## Introduction

In order for the immune system to clear viral infections, immune cells must be able to produce and respond to cytokines. During HIV infection, cytokine expression and functions become deregulated, contributing to broad immune dysfunction and disease progression. Interleukin-27 (IL-27) can function as a pro- or anti-inflammatory cytokine depending on cell type and activation status [1]. IL-27 is a member of the IL-12 family of cytokines, comprised of molecules sharing subunits and receptor chain components [2]. The IL-27 receptor (IL-27R) is heterodimeric, composed of the IL-27Rα subunit, called WSX-1, which is unique for the binding of IL-27, and a β receptor subunit, called gp130 [3]. The gp130 receptor chain is a commonly shared signaling receptor subunit for numerous other cytokines, including IL-6, oncostatin M (OSM), IL-11, leukemia inhibitory factor (LIF), cadiotrophin-1 (CT-1), cardiotrophin-like cytokine (CLC), ciliary neurotrophic factor (CNTF), and neuropoietin (NP) [4]. The WSX-1 receptor chain was identified as a result of sequence homology with the gp130 chain and, as such, is a characteristic type I cytokine receptor [5], [6]. Although IL-27 can bind with low affinity to WSX-1 in the absence of gp130, for effective signal transduction both IL-27R subunits must be expressed [3], [7]. A wide variety of cells respond to IL-27, as co-expression of the IL-27R subunits has been reported in endothelial cells, mast cells, activated B cells, monocytes, Langerhan’s cells, activated DCs, and T cells [3], [7], [8], [9], [10].

The IL-27 intracellular signaling pathways are well defined in terms of JAK/STAT activation. The WSX-1 subunit has a short cytoplasmic domain compared to gp130, but does have conserved tyrosine residues which impart the ability to activate JAK/STAT proteins [5]. Our previous work characterized a role for JAK/STAT signaling in mediating IL-27-induced activation of human monocytes, including upregulation of inflammatory responses like pro-inflammatory cytokine expression [11], [12].

IL-27 is a cytokine that is critical to the initiation of innate immune responses directed by monocytic cells and bridges to adaptive immunity by its influence on T cell differentiation. Thus, IL-27 can play a role in regulating inflammatory responses in monocytes/macrophages and CD4 T cells, both of which are primary targets of HIV infection. Interestingly, IL-27 can inhibit HIV replication in monocytes/macrophages and T cells, implicating IL-27 as a potent anti-HIV cytokine [13], [14]. Previously, we reported that clinical characteristics, including HIV viral load, hepatitis C virus coinfection, and CD4 T cell counts were associated with changes in serum IL-27 [15]. Herein, we further our previous findings and identify how IL-27 functions in the setting of HIV infection, including characterization of IL-27 receptor expression, and downstream functions of IL-27, such as induction of pro- and anti-inflammatory gene expression.

**Figure 1:**
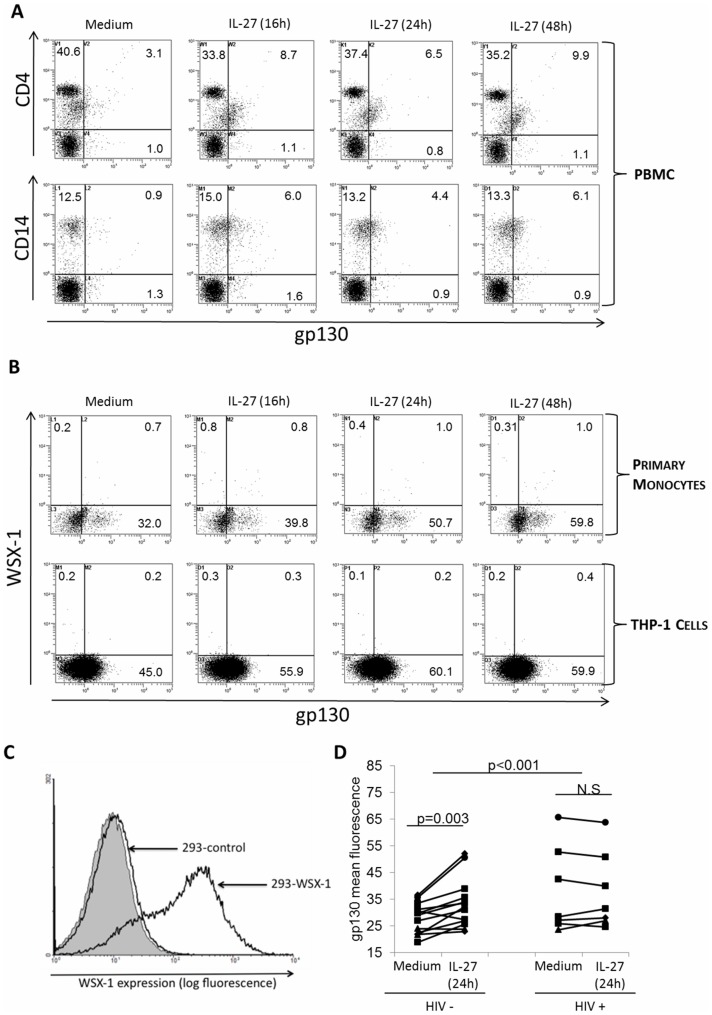
IL-27 induces surface expression of gp130 in monocytes from HIV negative individuals, and this response is impaired in monocytes from HIV positive individuals. PBMC from HIV-negative subjects were treated with a time course of IL-27 for 16, 24, and 48 h. (A) PBMC were co-stained with gp130 and CD4+ (top panel) or gp130 and CD14+ (bottom panel), and analyzed by flow cytometry. (B) Co-staining with gp130 and WSX-1 was performed on purified monocytes (top panel) and THP-1 cells (bottom panel). For (A) and (B), dot plots shown are annotated with percent of positive cells within each quadrant, and are representative of responses from 3 different blood donors and experiments. (C) Surface staining for WSX-1 was performed on HEK-293 cells transfected with WSX-1 (293-WSX-1) and cells transfected with a control cytokine receptor (293-control). Grey histogram represents autofluorescence of unstained cells. (D) PBMC from HIV negative (HIV−, n = 13), and HIV positive (HIV+, n = 7) subjects were stimulated with IL-27 for 24 h, and then surface stained for gp130. Values shown represent mean fluorescence intensity of gp130 staining in unstimulated (Medium) versus IL-27–stimulated (IL-27 24 h) PBMC within each group, with each joined data point representing one study subject. In the HIV+ group, one patient was included twice, as samples were obtained from study visits several months apart with different viral loads (study visit one: VL = 15, 532, MFI medium: 23.4 vs. MFI IL-27∶26.9; study visit two: VL = 137, 722, MFI medium: 52.7 vs. MFI IL-27∶50.7). For statistical analysis, within-group induction of gp130 expression was assessed separately using the paired samples t-test. To assess differences in IL-27-responsiveness of the HIV – versus the HIV + group, change scores were calculated, followed by between-group changes compared using the independent samples t-test.

## Methods

### Study Participants

#### Ethics statement

In accordance with Queen’s University Research Ethics Board approval, written informed consent was obtained from 13 HIV negative (controls) and 13 HIV positive, viremic, blood donors from the Clinical Immunology Outpatient Clinic (CIOC) at Hotel Dieu Hospital, Kingston, Ontario, Canada.

Due to limitations in the cell numbers collected per blood draw, not all assays could be performed on each sample. The number of patient samples completed for each analysis is included in the figures. Since three HIV positive patients had samples drawn at least 5 months apart with different viral loads at each visit, these were included twice (as indicated in figure legends) in some experiments to enhance statistical power. Viral load (VL, in copies/mL) and CD4+ T cell counts (cells/µL) were obtained during routine clinic visits at the CIOC. All subjects included in the HIV positive group were viremic, with a mean plasma viral load of 19086 (330–74912) copies/mL and CD4 T cells counts were 344 (30–771) cells/µL. Of the 13 HIV-positive study subjects, six were receiving HAART but still presented with high viral loads. However, of these patients, 3 were tolerant/non-adherent to HAART, 2 were two-class resistant (nucleoside reverse transcriptase inhibitors (NRTI) and non-nucleoside reverse transcriptase inhibitors (NNRTI)), and 1 was one-class resistant (NRTI).

**Figure 2:**
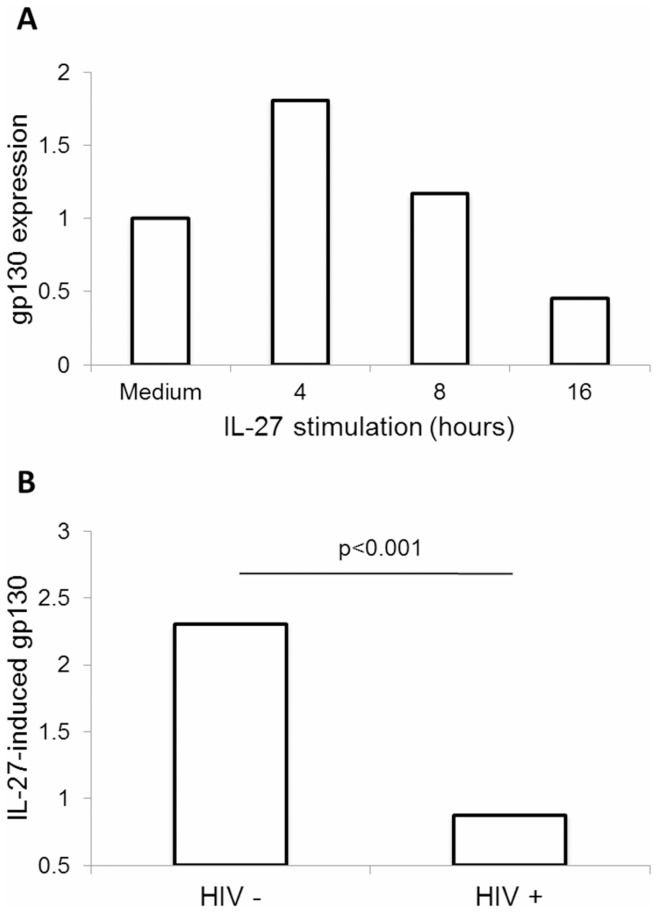
IL-27-induced mRNA expression of gp130 is impaired in HIV infection. (A) Purified blood monocytes were stimulated with IL-27 for 4, 8, and 16 h, then analyzed by real-time PCR for mRNA expression of gp130 (n = 4). (B) Monocytes from HIV negative (n = 8) and HIV positive subjects (n = 7) were stimulated for 4 h with IL-27, and then analyzed by real-time PCR for gp130 mRNA expression. Comparison of gp130 expression between the two groups was performed with an independent samples t-test. Data shown have been normalized to 18SrRNA expression, and represent IL-27-mediated induction of gp130 expression relative to expression levels in unstimulated cells (Medium).

### Cell Culture and Monocyte Isolation

THP-1 cells (pro-monocytic leukemic cells), were obtained from ATCC. Cells were cultured in Iscove’s Modified Dulbecco’s Medium (Gibco) supplemented with 10% Fetal Bovine Serum (Gibco). A HEK-293 T cell line constitutively expressing WSX-1/TCCR (293-WSX-1) and a control cell line expressing an alternative cytokine receptor (293-control) were gifts from Dr. Nathalie Arbour (Université de Montréal, Montreal, Canada) and Dr. Jean-François Gauchat (Université de Montréal, Montréal, Canada), and were used to confirm WSX-1 flow cytometry staining, as previously described [16]. Peripheral blood mononuclear cells (PBMC) were isolated by blood overlays on Lympholyte (Cedarlane Laboratories) and subsequent density centrifugation. The PBMC fraction was processed for monocyte enrichment (Stemcell Technologies cat #19058) or cryopreserved in fetal calf serum + dimethyl sulfoxide (10%) at −80°C until future use. Purified monocytes were stimulated with recombinant IL-27 (120 ng/mL, R&D Systems) for 4 h, and then processed for real-time PCR. Frozen PBMC of all study participants were thawed simultaneously in IMDM with 20% FCS for 24 h in 24-well tissue culture plates. This was followed by removal and replacement of media (IMDM, 10% FCS) and subsequent stimulation with IL-27 for 24 h. Cells were harvested and used in flow cytometry, while culture supernatants were assayed by ELISA.

### RNA Isolation and Real Time-PCR

Total RNA was extracted from monocytes using TRI-Reagent. RNA quantification was performed using the ND-1000 (NanoDrop Technologies) spectrophotometer. RNA (1 µg) was reverse transcribed using the Moloney Murine Leukemia Virus reverse transcriptase enzyme (Invitrogen). Template cDNA was used to measure gp130 expression (normalized to 18SrRNA) in cells treated with IL-27 for 4 h, relative to expression levels in untreated cells. Amplification was performed with gp130-specific primers, as previously described: 5′-TCTGGGAGTGCTGTTCTGCTT-3′ and 5′-TGTGCCTTGGAGGAGTGTGA-3′ [17], and 18SrRNA primers: 5′-ACT-CAA-CAC-GGG-AAA-CCT-CAC-C-3′, 5′-CCA-GAC-AAA-TCG-CTC-CAC-CAA-C-3′ (IDT DNA Technology). Real-time PCR cycling was performed using the SsoFast EvaGreen Supermix (Bio-Rad) on the Rotor-Gene 6000 (Corbett Life Sciences).

**Figure 3:**
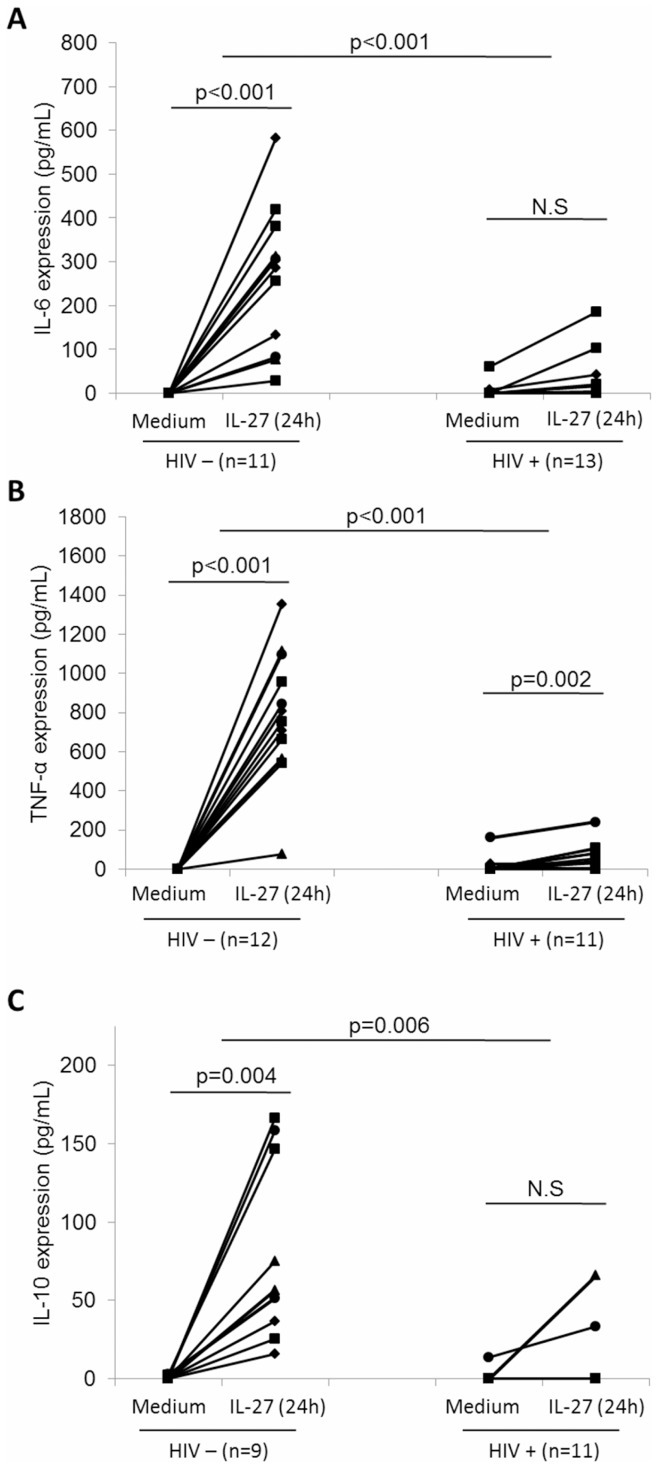
IL-27-induced cytokine production is downregulated in HIV infection. Supernatants from PBMC cultured in the presence or absence of IL-27 for 24 h (1×106 cells/mL) were used in ELISA to quantify IL-27-induced cytokine production. (A) IL-6, (B) TNF-α, and (C) IL-10 expression are shown, with each set of connected data points representing one subject before and after IL-27 stimulation. Sample sizes are annotated under each group. Statistical analyses of within-group and between-group cytokine induction were performed with a paired samples t-test and independent samples t-test respectively.

### Flow Cytometry

PBMC were harvested and washed in PBS-azide (PBS +0.1% azide and 1% BSA) then stained with fluorochrome-conjugated antibodies: gp130-FITC and TCCR/WSX-1-PE (R&D Systems), CD14-PE (Beckman Coulter), and CD4-PECy5 (eBioscience). After staining, cells were washed with PBS-azide, and data were acquired with the Epics XL-MCL flow cytometer. Data were analyzed using the WinMDI version 2.9 software package (J. Trotter, Scripps Institute, San Diego). Live cells were gated in all analyses.

### ELISA

Culture supernatants were used to quantify cytokine expression as previously described [12] and according to manufacturer instructions: IL-6 (Biosource), TNF-α (Biosource), and IL-10 (eBioscience). Absorbencies were measured with the BioTek ELx800 Microplate Reader (Fisher Scientific). Individual patient sera were assayed in duplicate and the averages are reported in this study.

### Statistical Analysis

The Medical Research Centre at Queen’s University provided expertise in analyzing patient data. Comparison of IL-27-induced functions in HIV negative versus HIV positive groups was assessed by the independent samples t-test. Additionally, to assess responses to IL-27 within groups (HIV negative or HIV positive) the paired samples t-test was used.

## Results

### IL-27 Induces Expression of gp130, but not the Counterpart Receptor Subunit, WSX-1, in HIV-negative Monocytes

The IL-27 receptor is heterodimeric, composed of gp130 and WSX-1 subunits [3]. It is not known whether IL-27 stimulation influences expression levels of the IL-27R. Therefore, we examined the possibility that IL-27 treatment could enhance either gp130 or WSX-1 expression in PBMC. Interestingly, we observed that a time course of IL-27 treatment resulted in induction of gp130 surface expression on the monocyte population within PBMC (Fig. 1A). More specifically, we observed two populations of CD4+ stained cells (T cells and monocytes), with only one population responding to IL-27 via upregulation of gp130 (Fig. 1A, top panel). Furthermore, we confirmed that this population was indeed monocytes by gating on CD14+ cells (Fig. 1A, bottom panel). Since we identified that IL-27 could induce gp130 expression, we next examined whether IL-27 could upregulate WSX-1 expression. Purified primary monocytes were treated with a time course of IL-27 and co-stained with gp130 and WSX-1 (Fig. 1B, upper panel). As expected, we observed a significant upregulation of gp130 expression from IL-27 treatment: an increase from 32% at baseline (Medium) to 59.8% of gp130 positive cells after 48 h of IL-27 treatment. These results were confirmed in the human monocytic cell line, THP-1, with an increase in gp130 expression from 45% to 59.9% upon IL-27 treatment (Fig. 1B bottom panel). In parallel, no increases in WSX-1 expression in either cell type were observed. As a positive control for WSX-1 detection, we performed WSX-1 staining on HEK-293 cells stably expressing the human WSX-1 gene (Fig. 1C).

### IL-27-induced Cell Surface Expression of gp130 is Impaired in HIV Infection

Since we identified gp130 as a novel IL-27-responsive gene, we decided to investigate if the ability of IL-27 to induce gp130 was altered in the setting of HIV-infection. PBMC from HIV negative (HIV −) and HIV positive (HIV +) subjects were stimulated with IL-27 for 24 hours, a time point which showed significant induction of gp130 expression in response to IL-27 in Fig. 1A and B. Following IL-27 stimulation cells were surface-stained for gp130 expression (Fig. 1D). We observed a significant increase in gp130 expression, as measured by mean channel fluorescence, in HIV negative subjects (p = 0.003), while no significant induction was observed in HIV positive subjects (p = 0.99). Additionally, when comparing the magnitude of the IL-27 response (fold change in gp130 expression) within the HIV − group to that of the HIV + group, we observed a significant difference (p<0.001) in responses of the two groups to IL-27, indicating impaired IL-27 responses in HIV infection. Interestingly, 2 of the 13 HIV+ individuals exhibited higher levels of basal gp130 expression compared to HIV− subjects, however, differences in basal expression of gp130 between HIV+ and HIV− groups was not statistically significant.

### IL-27-induced mRNA Expression of gp130 is Impaired in HIV Infection

To follow our findings at the cell surface with gp130, we investigated IL-27-induced gp130 expression at the mRNA level. We initially performed a short time course (4–16 h) of IL-27 treatment on primary monocytes to characterize the kinetics of IL-27-induced gp130 mRNA expression by real-time PCR. We observed gp130 induction at 4 h of IL-27 treatment, in primary monocytes (Fig. 2A). To observe the effect of HIV infection on IL-27-induced mRNA expression of gp130, we stimulated monocytes from HIV negative and HIV positive individuals for 4 h with IL-27, and subsequently compared the induction of gp130 mRNA expression (Fig. 2B). Results shown represent fold increase in gp130 expression from 4 h of IL-27 treatment compared to untreated cells from the same subject, normalized to 18SrRNA expression. We observed a significant decrease in IL-27-induced mRNA expression of gp130 in the HIV-positive group compared to the HIV-negative group. Values for the two groups were compared using an independent samples t-test, with p<0.001.

### IL-27-induced Cytokine Production is Downregulated in HIV Infection

To further identify the effect of HIV infection on IL-27 function, we performed cytokine ELISAs on supernatants from PBMC treated for 24 h with IL-27. We looked at previously described IL-27-responsive cytokines: pro-inflammatory cytokines IL-6 and TNF-α, and the anti-inflammatory cytokine IL-10 (Fig. 3) [12], [18]. We observed significant differences in IL-27-induced cytokine production between groups, with the HIV positive group showing consistently decreased IL-27-mediated cytokine induction compared to the HIV negative group. Furthermore, within the HIV positive group itself, no significant induction of IL-6 or IL-10 was observed in response to IL-27 stimulation, indicating suppression of IL-27 function. Interestingly, a significant induction of TNF-α was observed within the HIV positive group in response to IL-27 (p = 0.002), indicating that the cells are still responsive to IL-27. However, the magnitude of induction in response to IL-27 in the HIV positive group was significantly less than that of IL-27-induced TNF-α in the HIV negative group. Thus, overall, we observed that IL-27-induced production of IL-6, TNF-α, and IL-10 is downregulated in HIV infection.

## Discussion

IL-27 is a critical cytokine that plays a role in the balance of inflammatory immune responses and CD4 T cell development. In this study we observed impairment of IL-27-induced gene expression, including downregulation of IL-27-induced gp130 mRNA and surface protein expression, and downregulation of IL-27-induced cytokine production. These dysregulated IL-27 functions in HIV infection represent novel mechanisms that may contribute to the widespread immunologic dysfunction observed in HIV infection.

Deregulation of cytokine receptor expression during HIV infection has been previously demonstrated in lymphoid and myeloid cells [19], [20], [21]. Currently, no studies have investigated how HIV affects expression of the unique IL-27 receptor subunit, WSX-1. However, some studies have shown dysregulated expression of other related subunits. For example, dysregulated IL-12 receptor (homologous to gp130) expression has been detected in the setting of HIV infection [19], [20] and common variable immunodeficiency [22], whereas upregulated gp130 expression has been detected in lymphoid tissues of HIV-infected patients at different stages of disease progression [21]. Interestingly, this study identified the IL-27 receptor chain, gp130, as a novel IL-27-responsive gene in human monocytes. Furthermore, we observed downregulation of IL-27-induced gp130 expression at both the mRNA and surface protein level in HIV positive subjects compared to HIV negative subjects. We found surface expression of WSX-1 to be undetectable; it is possible that this is due to very low expression levels of this receptor subunit, below the threshold of detection, yet still enough to mediate IL-27 responsiveness. Indeed, monocytes are responsive to stimulation with IL-27, as demonstrated in the IL-27-mediated induction of gp130 expression (Fig. 2A) and cytokine induction (Fig. 3) observed in HIV negative individuals. Furthermore, previous work from our lab has demonstrated that IL-27-mediated cytokine induction is regulated by STAT1, STAT3, and NF-κB induction in primary human monocytes [11], [12].

Our findings revealed a defect in IL-27-induced transcriptional responses, resulting in impaired inducible receptor expression on the cell surface. Indeed, further study is required to dissect the precise deficiencies in IL-27 signaling that result in downregulated gene expression, in particular with the examination of STAT activation. In line with this, previous work in cells isolated from HIV positive blood showed a disconnect between cytokine stimulation and gene expression, reporting differences in STAT1-dependent gene expression and STAT1 phosphorylation [23]. In view of the role played by IL-27 signaling in CD4 T cell development as well as in monocyte activation, further studies in this regard will be critical to defining which IL-27-induced signaling pathways, including that of STAT1 and STAT3, are altered during HIV infection.

It is well established that HIV infection causes dysregulated cytokine production, resulting in impaired immune responses, characteristic of HIV infection and progression to acquired immunodeficiency syndrome (AIDS). More specifically, in the setting of HIV-infection, monocytes/macrophages have exhibited enhanced pro-inflammatory cytokine production, with increased basal levels of IL-1, IL-6, IL-8, and TNF-α [24], [25], [26]. Interestingly, contradictory results on how HIV-infection affects pro-inflammatory cytokine production have also been reported [27]. A previous study investigated expression levels of MIP-1α, RANTES, IL-8, IL-1α, IL-3, IL-6, GM-CSF, G-CSF, TNF-α, TGF-β1 in the setting of *in vitro* infected macrophages, and found only MIP-1α production to be enhanced. Herein, in addition to assessing basal cytokine production, we measured IL-27-induced cytokine production, as a measure of IL-27 function. Accordingly, levels of IL-27-induced IL-6, TNF-α, and IL-10 were significantly downregulated in the HIV positive subject group compared to the HIV negative group, indicating impairment of IL-27-induced cytokine expression in the setting of HIV infection. Interestingly we observed IL-27-induced TNF-α to be significant in the HIV positive group, albeit at a lesser magnitude of induction than in the HIV negative group. This indicates that IL-27 function is not completely impaired. Indeed it is possible that other IL-27-mediated effects may not be affected by HIV infection; IL-27 has been shown to modulate TLR4 surface expression as well as MHC expression in human monocytic cells [11], [28]. Further study into the effect of HIV infection on these and other IL-27 functions, including that on CD4 T cell development, will be critical to advancing our understanding of this key cytokine in HIV infection.

Pertinent to our study, Tilton *et al.* (2006) reported diminished pro-inflammatory cytokine production in patients with high level viremia experiencing an interruption of ART, and showed an inverse relationship between cytokine production and type I IFN (IFN)-stimulated gene activation [29]. This observation suggests elevated IFN-stimulated gene expression occurs in HIV-viremic subjects off ART, resulting in activated immune cells that are impaired in their ability to upregulate proinflammatory cytokine expression. All HIV positive individuals in our study were viremic and included six individuals who were receiving HAART but were classified as treatment-resistant, and consequently presented with high viral loads. We observed the same trend of reduced IL-27 responsiveness in all of our patients, including those receiving HAART. Considering that only six individuals were receiving HAART and still had high viral loads, it is likely that the virus, and not HAART, which causes the observed lack of response to IL-27. Further investigation using cells isolated from patients with HAART-controlled virus levels may demonstrate restoration of IL-27 function. Data from these experiments will aid in identifying if HIV can directly impact IL-27 function or expression. It is also possible that other cytokines may be involved in the regulation of IL-27 function; interestingly, previous studies have reported high levels of IFN in HIV-infected patients with advanced infection [30], and, furthermore, it has been reported that IL-27 can induce IFN expression [13]. However, our previous data suggested that circulating IL-27 levels may be repressed in HIV positive individuals with high viral load [15]. Thus, further studies in this regard are needed to clarify the relationship between IFN and IL-27, and will be important to our understanding of the complex role played by these cytokines in HIV pathogenesis.

In conclusion, this study characterizes the effect of HIV infection on IL-27 function. In particular we demonstrate that levels of the IL-27 receptor chain, gp130, are decreased in response to IL-27. Our data showing that IL-27-induced cytokine production is compromised in HIV positive subjects is suggestive of immune cell dysregulation and is particularly important to future studies on how HIV impacts IL-27 function in other arms of the immune system, including that of Th cell development.
